# DEK Expression in Breast Cancer Cells Leads to the Alternative Activation of Tumor Associated Macrophages

**DOI:** 10.3390/cancers12071936

**Published:** 2020-07-17

**Authors:** Nicholas A. Pease, Miranda S. Shephard, Mathieu Sertorio, Susan E. Waltz, Lisa M. Privette Vinnedge

**Affiliations:** 1 Division of Oncology, Cancer and Blood Diseases Institute, Cincinnati Children’s Hospital Medical Center, Cincinnati, OH 45229, USA; peasen@uw.edu (N.A.P.); Miranda.Shephard@cchmc.org (M.S.S.); Mathieu.Sertorio@cchmc.org (M.S.); 2 Molecular and Cellular Biology Program, Department of Bioengineering, University of Washington, Seattle, WA 98105, USA; 3 Department of Pediatrics, University of Cincinnati College of Medicine, Cincinnati, OH 45229, USA; 4 Department of Cancer Biology, University of Cincinnati College of Medicine, Cincinnati, OH 45267, USA; waltzse@ucmail.uc.edu; 5 Research Service, Cincinnati Veterans Affairs Medical Center, Cincinnati, OH 45267, USA

**Keywords:** DEK, tumor microenvironment, tumor associated macrophages

## Abstract

Breast cancer (BC) is the second leading cause of cancer deaths among women. DEK is a known oncoprotein that is highly expressed in over 60% of breast cancers and is an independent marker of poor prognosis. However, the molecular mechanisms by which DEK promotes tumor progression are poorly understood. To identify novel oncogenic functions of DEK, we performed RNA-Seq analysis on isogenic Dek-knockout and complemented murine BC cells. Gene ontology analyses identified gene sets associated with immune system regulation and cytokine-mediated signaling and differential cytokine and chemokine expression was confirmed across Dek-proficient versus Dek-deficient cells. By exposing murine bone marrow-derived macrophages (BMDM) to tumor cell conditioned media (TCM) to mimic a tumor microenvironment, we showed that Dek-expressing breast cancer cells produce a cytokine milieu, including up-regulated Tslp and Ccl5 and down-regulated Cxcl1, Il-6, and GM-CSF, that drives the M2 polarization of macrophages. We validated this finding in primary murine mammary tumors and show that Dek expression in vivo is also associated with increased expression of M2 macrophage markers in murine tumors. Using TCGA data, we verified that DEK expression in primary human breast cancers correlates with the expression of several genes identified by RNA-Seq in our murine model and with M2 macrophage phenotypes. Together, our data demonstrate that by regulating the production of multiple secreted factors, DEK expression in BC cells creates a potentially immune suppressed tumor microenvironment, particularly by inducing M2 tumor associated macrophage (TAM) polarization.

## 1. Introduction

The prognosis of patients with breast cancer is strongly influenced by the non-cancer cells in the microenvironment. In addition to the cancer cells themselves, several other cell types are present in the tumor microenvironment, including endogenous non-neoplastic cells, endothelial cells, cancer associated fibroblasts, and a complex array of infiltrating leukocytes [1]. While lymphoid-derived cells, such as CD8+ cytotoxic T cells, may participate in immune surveillance by suppressing malignant cell growth, myeloid-derived cells have documented roles in promoting tumor development and metastasis [2,3,4]. Macrophages, in particular, are abundant in solid tumors and can serve as benevolent immune cells in the tumor microenvironment. Clinical data reveal a negative correlation between tumor-associated macrophage (TAM) density and patient survival as well as response to therapy in all subtypes of breast cancer [5,6,7,8]. Nonetheless, the activation of these macrophages ultimately dictates their function within the tumor microenvironment. The activation of macrophages occurs when they are exposed to small molecules and cytokines or chemokines produced by other cells in the microenvironment, which allows a fine-tuned response to environmental cues. Depending on the stimulus, activated macrophages are sub-divided by phenotype into either classically activated (M1) and alternatively activated (M2). M1 activation, which is largely seen as tumor-inhibiting, is characterized by enhanced phagocytosis function as well as nitric oxide, IL-6, IL-12, and TNF production [9]. M2 activation, which is considered to be a tumor-promoting state, is characterized by the production of arginase, IL-10, and specific membrane proteins like MRC1 and CD163 [9]. There exists a spectrum of M2-like activation states that are characterized by a variety of sensor and effector molecules associated with tissue repair and immune suppression [9,10,11]. Importantly, M2-like TAMs release immunosuppressive cytokines, such as IL-10 and TGFb, and express programmed cell death 1 (PD-L1) to inhibit T cell function in the tumor microenvironment [12]. Furthermore, M2-like macrophages recruited to the perivascular sites within the tumor dramatically enhance angiogenesis, in part through upregulated production of VEGFa, and promote tumor cell intravasation in mammary tumors [13,14,15,16]. Given the potential advantages macrophages provide in tumor development and metastasis, there is a need to better understand the mechanisms of macrophage activation and plasticity, and their downstream effects in the tumor microenvironment.

Previously, our work and the work of others’ have shown that the chromatin remodeling DEK protein is over-expressed in >60% of all breast cancer cases and is a marker of highly proliferative tumors with poor prognosis [17,18]. DEK also is overexpressed in many solid tumors including hepatocellular carcinoma, bladder cancer, cervical cancer, prostate cancer, colon cancer, and head and neck squamous cell carcinomas [18,19,20,21,22,23,24,25]. Transcriptional up-regulation of *DEK* by an activated Rb/E2F pathway, or YY1, NF-Y, and ER-α transcription factors, are frequently the causes of DEK overexpression in these solid tumors [26,27,28]. Importantly, high DEK expression correlates with more aggressive and chemoresistant tumors, but the mechanisms underlying these characteristics are poorly understood [27,29]. While the cell intrinsic effects of oncogenic DEK expression have been explored, the cell extrinsic consequences remain understudied. For example, DEK has repeatedly been shown to fine-tune RelA/NFkB p65 transcriptional activity, whose target genes include a long list of cytokines and chemokines with immune-modulating functions [30,31,32,33,34]. However, the functional consequences of NFkB deregulation by DEK, and the impact of the differentially expressed cytokines and chemokines on the tumor microenvironment, are unexplored. Therefore, this work sought to explore the transcriptional impact of DEK over-expression in breast cancers and the downstream consequences to the tumor microenvironment. We hypothesized that one mechanism of DEK-mediated tumor progression would be the creation of a pro-tumorigenic microenvironment that would impact the anti-tumor immune response.

DEK is an abundant, highly conserved phosphoprotein with documented roles in autoimmune diseases and solid tumor progression [32,35]. Although DEK has no known enzymatic activity, it has three DNA binding domains and is implicated in DNA replication and repair, transcription regulation, and chromatin remodeling [36,37,38,39]. With regards to transcription and chromatin remodeling, DEK interacts with some proteins, such as Daxx and HDACII, to repress target genes [40,41]. Alternatively, DEK also interacts with other proteins, including C/EBPa and AP-2a, to promote target gene activation [42,43]. In addition to interacting with transcription factors, DEK is also a histone chaperone that regulates the balance of histone H3.1 versus H3.3 distribution across the genome [37,44,45]. Importantly, the chromatin remodeling functions of DEK counteract replication stress and facilitate both homologous recombination (HR) and non-homologous end-joining (NHEJ) DNA repair [36,46,47]. Finally, increasing evidence also has demonstrated that DEK facilitates intron removal during mRNA processing and splicing [48,49,50]. Combined, it is evident that DEK has a complex molecular function that controls the topology of nucleic acids and regulates their organization and utilization by the cell. How these molecular functions translate to oncogenic consequences in solid tumors remains to be explored.

We have previously reported that expression of the mouse DEK gene (“Dek”) in the MMTV-Ron murine model of breast cancer supports tumor growth and metastasis, as determined by comparing MMTV-Ron/Dek^+/+^ and MMTV-Ron/Dek^−/−^ animals [51]. Herein, we expand upon our prior studies through an analysis of tissues and breast cancer cell lines generated from this model for the purpose of better understanding how Dek drives breast cancer progression in vivo. Since Dek is known to regulate chromatin organization and transcription, we hypothesized that Dek expression in breast cancer cells would result in transcriptionally deregulated genes to drive tumor progression. Transcriptomic analyses of isogenic cell lines show that Dek expression preferentially regulates the expression of cytokine and chemokine genes in breast cancer cells, along with other genes known to promote tumor progression. Using in vitro models, we demonstrate DEK expression in cancer cells promotes the M2-like polarization of bone marrow derived macrophages (BMDM). In vivo, we demonstrate in both this clinically relevant murine model and in human breast cancers that DEK expressing tumors regulate a similar pattern of immunomodulatory genes identified in our ex vivo cell culture model and contain CD163+ M2-like TAMs. Together, this is the first report identifying an oncogenic role for DEK expression within cancer cells through a mechanism involving the regulation of the tumor microenvironment and the tumor-immune response.

## 2. Results

### 2.1. Dek Expression Dictates the Differential Expression of Immune Genes

**Figure 1:**
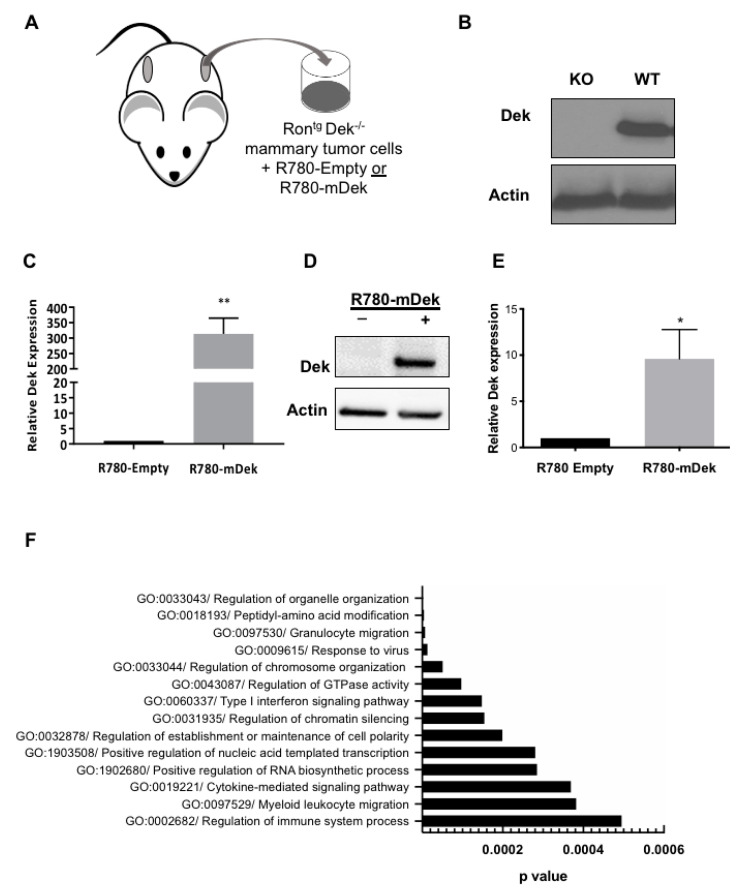
Transcriptomic profile of Dek expressing murine mammary cell lines reveals that genes controlling the immune response are de-regulated. (**A**) Schematic of establishing primary cells from Ron transgenic (Ron^tg^) Dek knockout (Dek^−/−^) mammary tumors and creating isogenic cell lines transduced with either R780-Empty or R780-mDek retroviral vectors. (**B**) Western blot of cells from Ron^tg^ Dek knockout (KO) and Dek wild-type (WT) mammary tumors. (**C**) Quantitative RT-PCR shows increased Dek expression in the Ron^tg^ Dek^−/−^ cancer cell line, RD147, transduced with murine Dek over-expressing constructs (R780-mDek) compared to empty vector (R780-Empty). (**D**) Western blotting shows restoration of Dek protein levels in RD147 R780-mDek cells compared to controls. (**E**) Quantification of Dek Western blot protein levels by densitometry. For mRNA and protein data, *n* = 3. * indicates *p*-value < 0.05 and ** indicates *p*-value is < 0.01 using Student’s *t*-test. (**F**) Selected gene ontology results using ToppFun based on RNA-Sequencing results for up- and down-regulated genes in RD147 R780-mDek cells compared to R780-Empty controls.

Previously, we described a murine breast cancer model with differential expression of the Dek oncogene [51]. This was accomplished by breeding the MMTV-Ron transgenic mouse model into a wildtype or germline Dek knockout background (Figure 1A,B) [51,52,53]. As previously reported, the Dek knockout mice were slower to develop mammary tumors that were also less metastatic. We subsequently generated multiple murine breast cancer cell lines on the FVBN genetic background that were deficient for Dek, then re-expressed murine Dek with a retroviral construct to create an isogenic model system (Figure 1C–E) [51].

To identify potential molecular mechanisms driving enhanced disease progression observed in Dek-expressing mammary tumors, we performed RNA sequencing analysis on a cell line created from Ron-Dek animal 147 (RD147), transduced with empty vector (R780-Empty) or a murine Dek cDNA (R780-mDek; Figure 1C–E). We identified 332 upregulated and 192 down-regulated genes in cells expressing the Dek oncogene compared to controls (Table S1). We then performed gene ontology analyses for the differentially expressed genes. In addition to known functions for Dek, such as “Regulation of chromosome organization,” gene ontologies related to immune system function were observed. These categories for differentially expressed genes included “Cytokine-mediated signaling pathway” and “Myeloid leukocyte migration,” among others (Figure 1F). Examples of differentially expressed genes for cytokines and other immune system-associated signaling molecules, like *Tslp, Ccl5, Nfkb2 (p52), Cxcl1, Cxcl2, Cxcl10, Ccl17,* and *Ccl25*, as well as other genes, such as *Vegfa* and *Cdh1*, that were previously described to be deregulated with Dek expression are listed in Table 1 [51,54,55,56].

**Figure 2:**
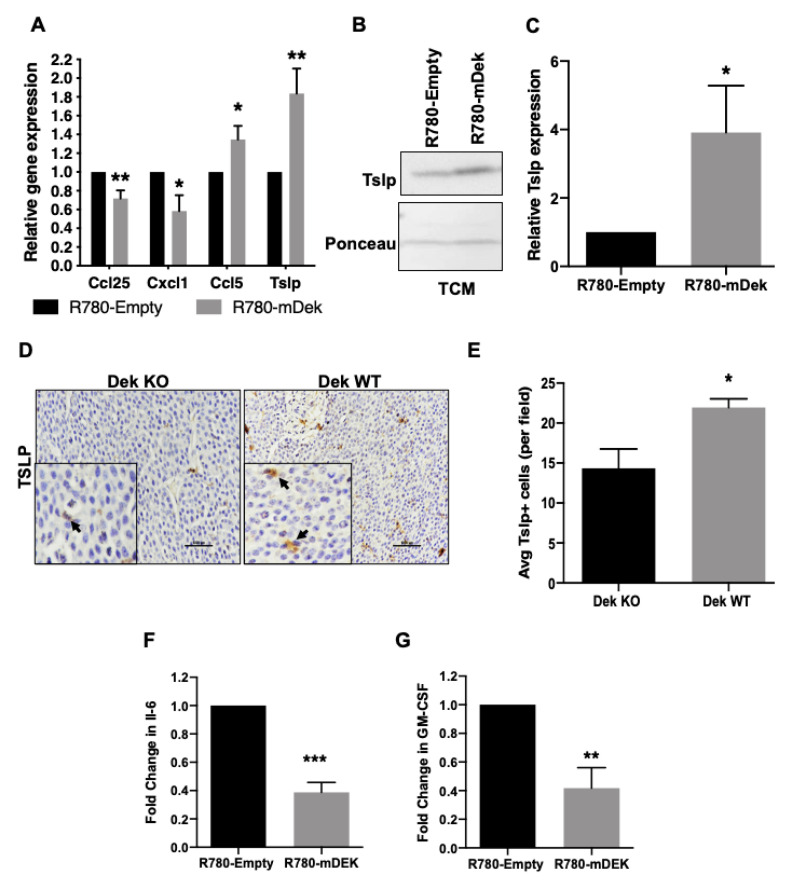
Cytokines associated with macrophage polarization and tumor-immune responses are deregulated with Dek expression. (**A**) mRNA levels of *Ccl25* and *Cxcl1* are down-regulated while *Ccl5* and *Tslp* are upregulated in Dek expressing RD147 cells as determined by quantitative RT-PCR (*n* = 3). (**B**) Western blot analysis shows Tslp protein is upregulated and secreted into tumor conditioned media (TCM) in Dek expressing cells and the quantitative densitometry is depicted in (**C**) *n* = 3. (**D**) Immunohistochemistry staining of Tslp was performed on primary mammary tumors from Ron^tg^ Dek KO (left) and WT (right) mice. (**E**) Quantification of (**D**), showing increased numbers of Tslp positive cells per field in Dek WT tumors compared to KO tumors, *n* = 6 tumors from different mice per genotype. (**F,G**) The downregulation of IL-6 (**F**) and GM-CSF protein (**G**) in R780-mDEK cells compared to R780-Empty cells was determined by a Luminex immunoassay. *n* = 3 independently generated cell lines (RD147, RD219, RD238). * indicates *p*-value < 0.05, ** indicates *p* < 0.01 and *** indicates *p* < 0.00 using Student’s *t*-test.

We then performed quantitative RT-PCR to confirm differential expression of several candidate cytokine and chemokine genes in Dek-expressing breast cancer cells identified by RNA-Sequencing. We confirmed transcriptional deregulation of genes including down-regulation of *Ccl25* and *Cxcl1* and up-regulation of *Ccl5* and *Tslp* (Figure 2A). For further validation, we examined Tslp protein secretion by Western blot and found that it was present at higher concentrations in conditioned medium from cultured DEK expressing RD147+R780-mDek cells compared to empty vector (R780-Empty) controls (Figure 2B,C). Furthermore, analysis of tumor tissue from the MMTV-Ron:Dek^+/+^ (Dek WT) mice demonstrated increased immunohistochemical staining for Tslp compared to MMTV-Ron:Dek^−/−^ (Dek KO) tumor tissue (Figure 2D,E). This indicates that the ex vivo cultured cells recapitulate gene expression differences observed in vivo.

We next sought to determine if additional cytokines were differentially expressed in Dek-expressing breast cancer cells that were not identified in the RNA-Seq analysis. We performed a Luminex multiplex immunoassay to assess the protein concentrations of additional cytokines secreted into conditioned media from cancer cell lines. We used previously published cells independently established from three different animals (RD147, RD219, and RD238) transduced with R780-mDek or empty vector [51]. While we did not observe consistent changes in the concentrations of Mcp-1 (Ccl2), IL-4, IL-13, S100A8, Lipocalin-2, or M-CSF (data not shown), both IL-6 and GM-CSF were consistently down-regulated in DEK-expressing cells compared to empty vector controls for all three cell lines (Figure 2F,G). The identification of cytokines in conditioned media devoid of cells, through both Western blotting (Figure 2B,C) and Luminex multiplex ELISA (Figure 2F,G), confirm that Dek-expressing cancer cells secrete different levels of cytokines compared to Dek-deficient cancer cells.

### 2.2. Dek Expression in Breast Cancer Cells Promotes M2-Like Macrophage Polarization In Vitro and In Vivo

**Figure 3:**
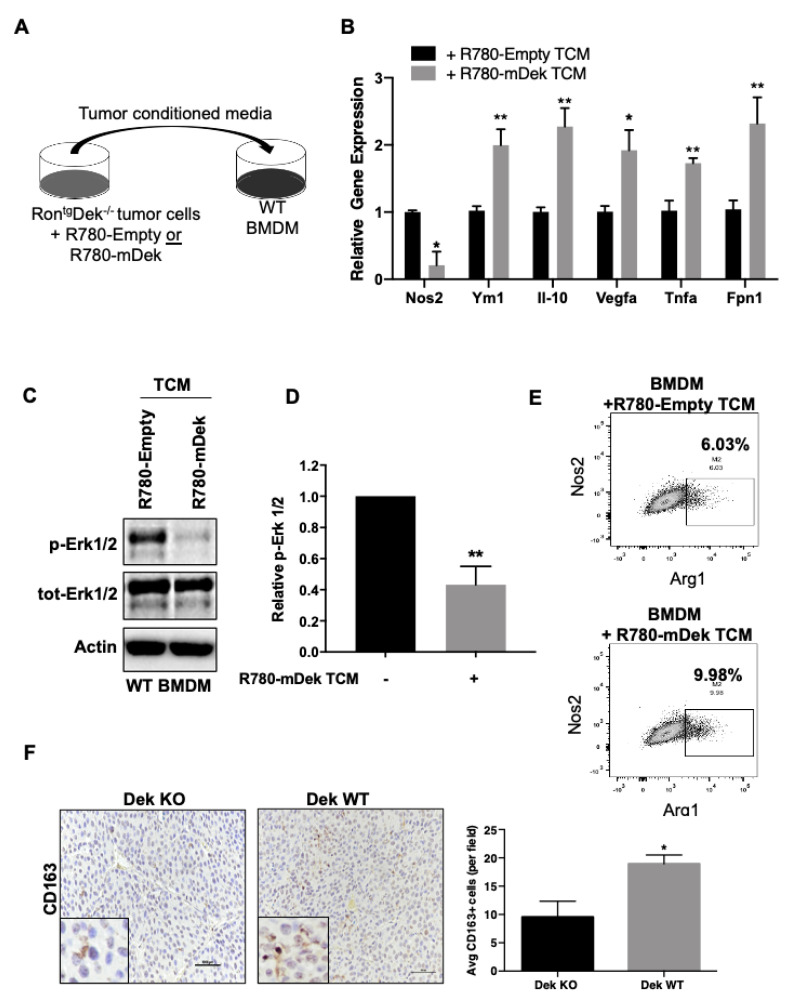
Bone marrow derived macrophages exposed to conditioned media from Dek-expressing breast cancer cells exhibit an M2 macrophage profile. (**A**) Schematic showing bone marrow derived macrophages (BMDM) from wild-type FVB/N mice were cultured in tumor conditioned media (TCM) from RD147 R780-Empty or R780-mDek cancer cells. (**B**) Quantitative RT-PCR shows down-regulation of the M1 marker *Nos2* and upregulated mRNA expression of various M2 macrophage markers in BMDM exposed to TCM from Dek-expressing cancer cells (“R780-mDek TCM”). *n* = 3–6 biological replicates each with technical duplicates. (**C**) Phosphorylated ERK1/2 protein expression is downregulated in BMDM exposed to R780-mDek TCM as determined by Western blotting and the densitometry results are graphed in (**D**), *n* = 3. (**E**) Flow Cytometry was performed on BMDM cultured in R780-Empty or R780-mDek TCM to examine the expression levels of NOS2 (M1 macrophage marker) and ARG1 (M2 macrophage marker). Flow data shows that BMDM exposed to R780-mDek TCM have more Arg1^hi^Nos2^lo^ cells (M2-like) compared to BMDM exposed to control Dek-deficient cells. (**F**) Immunohistochemical staining for CD163, an M2 specific macrophage polarization marker, shows a significant increase in the abundance of M2 macrophages in Dek WT tumors. The average number of positive CD163 cells were counted per field, as depicted in the graph on the right (*n* = 3 tumors from independent mice/genotype). * indicates *p*-value is <0.05 and ** indicates *p*-value is <0.01 using Student’s *t*-test.

Potential regulation of the anti-tumor immune response is a novel pro-oncogenic consequence of Dek over-expression. The cytokines identified in both RNA-Seq and the Luminex immunoassay could have significant consequences for tumor associated macrophage function. Specifically, the upregulated gene *Tslp* was previously demonstrated to facilitate the IL-4/IL-13 mediated alternative (M2-like) activation of macrophages and *Ccl5* is associated with the accumulation of TAMs [57,58,59]. However, down-regulated molecules in Dek expressing cells, such as GM-CSF, Ccl25, IL-6, and Cxcl1, are pro-inflammatory cytokines and known to promote M1-like activation of macrophages and their subsequent chemotaxis [60,61,62]. Therefore, we sought to determine if the Dek-mediated deregulation of secreted cancer-derived cytokines and chemokines had functional consequences and would impact the polarization of macrophages. To recreate an in vitro model of the tumor microenvironment, we isolated murine bone marrow derived macrophages (BMDM) from wild-type FVB/N mice and exposed them to cell-free tumor conditioned media (TCM) collected from RD147 R780-Empty or R780-mDek cells (Figure 3A). BMDM were then collected and analyzed for the expression of markers associated with M2 polarization and tumor associated macrophages. We found statistically significant increased mRNA expression of M2/TAM markers *IL-10, Vegfa, Tnfα, Fpn1*, and *Ym1*, as well as decreased expression of *Nos2* (“*iNOS*”) in BMDM exposed to conditioned media from Dek expressing cancer cells compared to controls (Figure 3B) [9,11]. Importantly, we saw no difference in response to TCM when comparing macrophages from Dek wild-type or knockout mice, suggesting this was a cell autonomous response to TCM (data not shown). We next investigated the intracellular signaling of BMDM exposed to TCM. Proinflammatory and classically activated M1 macrophages activate Erk1/2 signaling, while Erk1/2 signaling is not the primary mechanism for immunosuppressive M2 polarization [63,64]. Western blotting of lysates from BMDM exposed to TCM showed that Erk signaling was activated, as shown by phosphorylated Erk1/2, in BMDM exposed to TCM from Dek-deficient control cancer cells but was down-regulated significantly in TCM from Dek-expressing (R780-mDek) cells (Figure 3C,D). BMDM exposed to TCM from Dek-expressing cancer cells also increased the population of Arg1^hi^Nos2^lo^ macrophages compared to controls (Figure 3E). We next investigated the prevalence of M2 TAMs in primary murine mammary tumors from Dek knockout and Dek wild-type animals using CD163 as a marker. We noticed a prominent increase in the average number of CD163+ cells in Dek expressing tumors compared to Dek knockout tumors (Figure 3F).

### 2.3. Dek Expression in Breast Cancers Promotes Macrophage Polarization to an Iron-Recycling M2 Subtype

**Figure 4:**
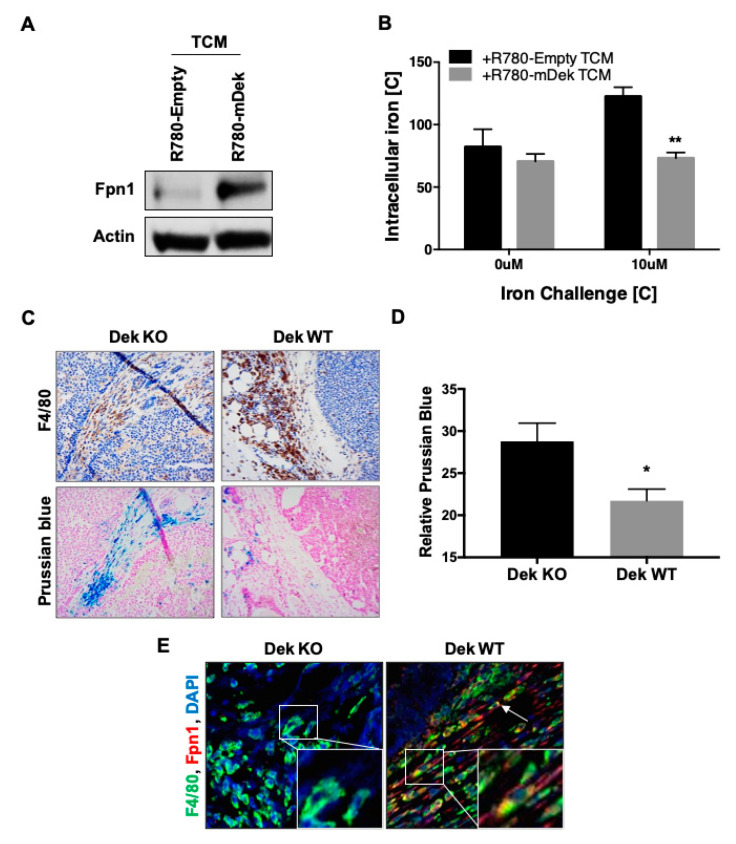
Macrophages exposed to Dek expressing cancer cells exhibit an M2-like iron recycling phenotype. (**A**) Ferroportin 1 (FPN1) iron transporter protein expression is upregulated in macrophages cultured in R780-mDek TCM compared to macrophages cultured in R780-Empty TCM. (**B**) BMDM exposed to TCM from RD147 Dek expressing cancer cells (R780-mDek TCM) retained less iron with the addition of 10 uM iron challenge compared to BMDM cultured in TCM from Dek-deficient R780-Empty cells, *n* = 3. (**C**) Immunohistochemistry was performed on adjacent sections of mammary tumor tissue from Dek KO (left) and WT (right) mice for a pan macrophage marker, F4/80, (top) and Perls Prussian Blue, a stain for ferric iron retained within cells (bottom). (**D**) Quantification of Prussian blue staining in Dek WT tumors was significantly less than seen in Dek KO tumors, as determined by ImageJ analysis, *n* = 3 per genotype. (**E**) Immunofluorescence was performed on Dek KO (left) and WT (right) murine mammary tumors for F4/80 (green) and Fpn1 (red) and DAPI (blue). White arrows in the Dek WT tumor indicated the co-localization of F4/80 and Fpn1. * indicates *p*-value is <0.05 and ** indicates *p*-value is < 0.01 using Student’s *t*-test.

Classically (M1) and alternatively (M2) activated macrophages are known to differ in their handling of iron transport and retention [65]. M1 macrophages retain iron to sequester it away from pathogens while IL-4-induced M2 macrophages recycle iron, sending it back into the microenvironment to support tissue repair and growth [66]. We next characterized how BMDM handled iron in response to exposure to TCM from control and Dek-expressing breast cancer cells. We first analyzed the expression of the only known cellular iron exporting protein, ferroportin (Fpn1). In line with mRNA results (Figure 3B), protein levels of ferroportin were significantly higher in BMDM exposed to TCM from Dek-expressing cancer cells (Figure 4A). We next tested the function of ferroportin in BMDM exposed to TCM by measuring the concentration of intracellular iron. Without excess iron added to culture media, BMDM exposed to TCM from control and Dek-expressing cancer cells had similar concentrations of intracellular iron. However, upon a challenge of excess environmental iron, BMDM exposed to TCM from Dek-expressing cancer cells had much lower intracellular concentrations of iron compared to BMDM exposed to TCM from Dek-deficient breast cancer cells (Figure 4B). This supports an iron-recycling M2-like phenotype for BMDM exposed to the microenvironment of Dek-expressing cancer cells. Subsequently, we examined whether this phenomenon occurred similarly in vivo utilizing a histological stain for intracellular iron, Perls Prussian Blue. We stained sequential sections of tissue from Dek knockout and Dek wild-type tumors with F4/80 (murine pan-macrophage marker) or Perls Prussian Blue for iron. The relative staining intensity of Perls Prussian Blue in areas that overlapped with F4/80 was significantly less in Dek wild-type tumors compared to knockout tumors (Figure 4C,D). To further support this, we co-stained tumors from Dek knockout and wild-type mice with F4/80 and ferroportin and found that only TAMs from Dek wild-type mice expressed ferroportin in vivo (Figure 4E). Combined, the data indicate there is an iron-recycling M2-like polarization of TAMs when exposed to the microenvironment of Dek-expressing cancer cells.

### 2.4. DEK Expression in Human Primary Breast Cancers Is Associated with Macrophage Regulation and Poor Survival

**Figure 5:**
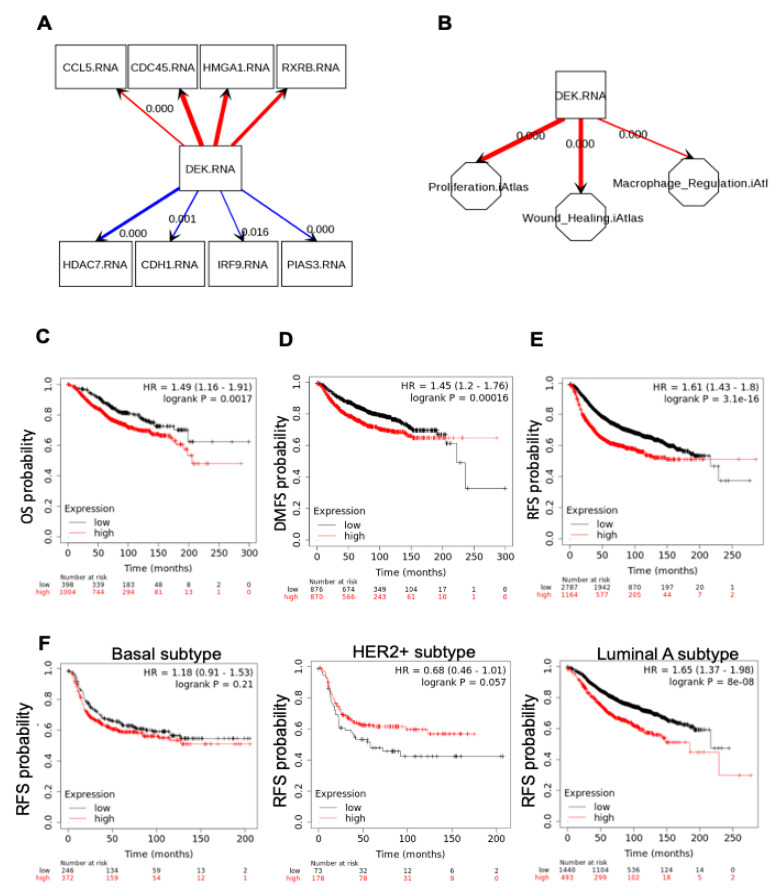
Gene expression profiles of Dek-expressing murine breast cancers are also detected in primary human breast cancers. SEMA software was utilized to plot correlations of expressed genes in breast cancers using data from The Cancer Genome Atlas (TCGA). (**A**) Genes that are up-regulated (positively correlated, top) and down-regulated (negatively correlated, bottom) with DEK expression in human breast cancer are depicted. Genes shown were selected from the list of genes in Table 1 that were found to be correlated in the mouse model. (**B**) A model of down-stream consequences of DEK expression based on gene expression correlations in primary human breast cancers that overlaps with the Dek-proficient and -deficient mouse model. SEMA software was used to query the association of DEK with cancer phenotypes in the Pan-Cancer Atlas. DEK expression positively correlates with proliferation, macrophage regulation, and a wound healing (M2-like) profile. For SEMA maps, red arrows depict positive correlations and blue arrows depict negative correlations. The thickness of the arrow represents the strength of the correlation, with *p* values shown when possible. If *p* values are not shown, then *p* < 0.0001. (**C–F**) KM plotter mRNA for breast cancer database was utilized to assess the association between DEK and CD163 expression on overall survival (**C**), distant metastasis-free survival (**D**), relapse free survival (**E**), and overall survival among different intrinsic molecular subtypes of breast cancer (**F**).

We next sought to determine if the correlations between DEK and a pro-tumorigenic immune microenvironment observed in our murine model also occurs in human patients. We utilized SEMA and The Cancer Genome Atlas resources to query correlations between DEK expression and genes identified in our RNA-Seq. We were able to validate multiple correlations with DEK expression and both down- and up-regulated genes in primary human breast cancers, including CCL5, CDC45, CDH1, and HDAC7 (Figure 5A). Furthermore, we observed a strong positive correlation between the expression of DEK and the M2 TAM marker CD163 (*p* = 0.005, data not shown) in primary breast cancers. We then used SEMA to query if DEK expression was associated with tumor phenotypes that we have identified in our Dek wild-type and knockout breast cancer model. Indeed, we confirmed a strong positive correlation between DEK and a previously identified tumor phenotype, proliferation. We also identified a novel association between DEK expression in human breast cancers with both a “wound healing” and a “macrophage regulation” phenotype that, when combined, support the findings in our mouse model of an M2 polarization of TAMs (Figure 5B).

We then investigated if the co-expression of DEK and CD163 (M2 macrophages) was associated with patient outcome. Breast cancer patients with gene expression signatures that were high for both DEK and CD163 showed decreased overall survival (OS), distant metastasis-free survival (DMFS), and especially relapse-free survival (RFS; Figure 5C). To look specifically at the strong association between high DEK/CD163 and RFS, we next queried RFS for various clinical and pathological variables. Interestingly, although high DEK/CD163 dual expression did not correlate with RFS in basal subtype or HER2+ breast cancer (Figure 5D,E), there was a strong correlation with decreased RFS in luminal A subtype (Figure 5F) and a similar correlation with luminal B subtype (data not shown).

## 3. Discussion

We previously published for the first time the role of Dek in a murine breast cancer model. In this Ron receptor tyrosine kinase transgenic model, with wild-type or knockout Dek alleles, we discovered that Ron(^tg^)/Dek^−/−^ mice were able to form mammary tumors but showed significantly delayed tumor initiation and fewer distant metastases when compared to Ron(^tg^)/Dek^+/+^ tumors [51]. To elucidate the mechanism(s) behind this difference in tumor growth kinetics and metastasis, we generated isogenic cell lines from primary murine breast cancers and performed RNA-Sequencing. While previously identified differentially expressed genes from prior publications were confirmed, such as E-cadherin down-regulation, several novel candidates were identified. Furthermore, gene ontology analyses indicated that, in addition to known functions such as transcriptional regulation and chromosome organization, a novel category of target genes, largely cytokines and chemokines, were identified that clustered with several immune-system related gene ontology categories. While DEK has been implicated in immune responses previously, particularly as an auto-antigen in autoimmune diseases and as a major component of neutrophil extracellular traps (NETs), its role in directing the tumor-immune response and the tumor microenvironment has never before been investigated [32,67,68]. Given that one of the gene ontologies from the RNA-Seq data specifically identified differentially expressed genes associated with myeloid leukocytes, we focused on how Dek expression within breast cancer cells may impact the polarization and function of tumor associated macrophages.

When exposed to stimuli within the microenvironment, macrophages within a tissue can polarize to a spectrum of activation states, from classically activated M1 macrophages to a variety of alternatively activated M2 states [11]. M1 macrophages are damaging to the tissue and inhibit tumor growth while M2 macrophages are wound-healing and tissue rebuilding macrophages that are considered tumor-promoting when located within the tumor microenvironment. We hypothesized that Dek expression within cancer cells would result in the expression and secretion of cytokines and chemokines that would drive macrophages towards an M2 polarization state to support tumor progression. We identified and confirmed the over-expression of genes known to induce M2 polarization and myeloid leukocyte migration, including *Tslp* and *Ccl5*, and the downregulation of genes that promote M1 polarization, including *GM-CSF, Ccl25,* and *Cxcl1* [57,58,59,62]. We then tested whether this shift in cytokine milieu had a functional impact on macrophage polarization. By exposing bone marrow-derived macrophages to conditioned media collected from tumor cells, we were able to confirm that Dek-expressing breast cancers direct an M2 polarization state compared to their Dek knockout counterparts. This was verified through a combination of the expression of M2 marker genes, the down-regulation of ERK1/2 signaling, and the acquisition of an iron recycling phenotype. These in vitro studies were further substantiated in primary murine mammary tumor tissue, through the co-staining of F4/80 with the iron transporter, Fpn1, the expression of the M2 marker CD163, and the loss of intracellular iron stores within TAMs as identified by Perl’s Prussian Blue staining. An iron recycling phenotype in TAMs is important because this provides an increased concentration of iron in the microenvironment to support the metabolic demands of rapidly proliferating tumor cells [65,69]. Finally, by using SEMA to visually depict gene expression correlations in data from TCGA, we confirm that our findings in the mouse model coincide with the impact of DEK expression in human primary breast cancers. Specifically, we showed that DEK expression in human breast cancers correlates with several differentially expressed genes identified by RNA-Sequencing in our murine model, and that DEK expression positively correlated with “wound-healing” and “macrophage regulation” tumor phenotypes. Interestingly, we saw the most substantial predictive impact of DEK and CD163 staining in luminal breast cancers. Previous reports have indicated that CD163+ TAMs are most commonly found in triple negative (typically basal subtype) breast cancer and predict poor prognosis [70]. Therefore, for luminal subtypes, the high expression of DEK may promote a unique CD163+ subset of luminal breast cancers, which will need to be evaluated in future studies. Combined, our data strongly indicate that the expression of DEK within solid tumors, particularly breast cancer, results in the differential expression of several cytokines and chemokines that create a pro-tumorigenic milieu to drive M2 polarization of tumor associated macrophages.

As previously mentioned, the murine mammary tumor model utilized in this work depends on the over-expression of the Ron receptor tyrosine kinase. We previously determined that activation of the Ron receptor, either through over-expression or HGFL-ligand binding, resulted in increased Dek expression [51]. Recently, it was reported that Ron expression in prostate cancer cells was associated with M2 macrophage polarization [71]. Thus, the work presented here strongly suggests that the induction of Dek expression in Ron-driven tumors is, at least partially, responsible for the subsequent M2 polarization of macrophages within the tumor microenvironment. However, DEK expression in humans is upregulated in response to other mitogenic signals, including steroid hormones and E2F proteins [26,27]. Therefore, DEK over-expression can likely create a pro-tumorigenic microenvironment in Ron-independent cancers as well.

The mechanism(s) by which DEK may be controlling the expression of several cytokine and chemokine genes is deserving of further investigation. One potential mechanism is through the ability of DEK to function as a transcriptional repressor of the p65/RelA transactivation subunit of the canonical NFkB pathway [30,31]. NFkB signaling in cancers is complex, but it is a largely pro-inflammatory pathway. We identified several NFkB target genes to be differentially expressed (largely down-regulated) with increased Dek expression, including *Ccl17, IL-6, Cxcl1, Ccl5*, and *Cxcl10* [72,73,74,75,76]. Furthermore, using RNA-Sequencing, we observed an upregulation in the expression of the noncanonical *Nfkb2* gene (the p100/p52 subunit) with high Dek expression [77]. Non-canonical NFkB2 signaling creates a slow, sustained activation of the pathway compared to rapid canonical signaling, but it has also been shown that p100 can sequester the canonical p50/p65 heterodimer in the cytoplasm in breast cancer [78]. Overall, the role of non-canonical NFkB2 in breast cancer pathogenesis is under-studied. However, NFkB2 may cooperate with Dek-mediated inhibition of canonical NFkB signaling to control cytokine and chemokine gene expression that drive tumor progression through effects on the microenvironment and the anti-tumor immune response.

While the work presented here focuses on the differential expression of genes that produce protein cytokines to direct TAM polarization, we did not investigate alternative mechanisms of polarization, such as small molecules. There is growing evidence that small molecules can also have an effect on TAMs. One differentially expressed gene in our RNA-Seq screen is sphingosine kinase 2 (Sphk2), which produces the lipid sphingosine-1 phosphate (S1P). S1P lipid is oncogenic as well, promoting tumor growth, metastasis, and angiogenesis [79]. Of interest, SPHK2 expression, and subsequent S1P production, in human breast cancer MCF7 cells has been shown to direct macrophage polarization to a pro-tumorigenic, and anti-inflammatory state, in vivo and in vitro [80,81]. The ability of S1P to negatively regulate inflammatory responses of macrophages and promote M2 polarization and TAM infiltration have also been demonstrated in other models [82,83]. Lactate is a second small molecule previously shown to promote M2 polarization of macrophages and metastasis in several breast cancer models [84,85]. Previous studies have demonstrated that over-expressing DEK in human immortalized keratinocytes and squamous cell carcinomas results in a significant increase in lactate production and subsequent accumulation in conditioned media as a result of metabolic reprogramming [86]. Therefore, DEK over-expressing epithelial cells may produce and secrete several small molecules, such as S1P and lactate, in addition to the protein cytokines investigated here, to promote the M2 polarization of TAMs to drive tumor progression.

The work presented here is the first description of how DEK expression in tumor cells can promote tumor progression via cell extrinsic mechanisms. Substantial work has previously demonstrated that DEK expression in cancer cells promotes proliferation through multiple pathways, including Wnt signaling, and is required for DNA damage repair [46,47,51]. However, we now add novel information that Dek expression in mouse and, potentially, human breast cancer cells regulates the production of several cytokines and chemokines. These cytokines can create a potentially pro-tumorigenic microenvironment through the M2 polarization of TAMs. Given that over 60% of primary breast cancers express high levels of DEK, this work provides a greater understanding for how these tumors may progress to late stage disease. Some studies have shown that M2 TAMs can inhibit CD8+ T cell infiltration, which lessens the efficacy of anti-PD1 immunotherapy [87]. Thus, future work in primary breast samples and immunocompetent models are needed to determine if DEK expression can predict immunotherapy response due to its impact on TAM polarization. Combined, this suggests that targeting DEK expression, or inhibiting its downstream targets, may be an effective therapy that would have both a cytotoxic effect on breast cancer cells and could reactivate the anti-tumor immune response.

## 4. Materials and Methods

### 4.1. Mice

Ron^tg^Dek^+/+^ and Ron^tg^Dek^−/−^ mice were generated as reported previously. Briefly, Dek^−/−^ mice were backcrossed into an FVB/N background and Dek^+/−^ females were bred to MMTV-Ron male mice of the same FVB/N background. MMTV-Ron Dek^+/−^ males were bred to Dek^+/−^ females to generate Ron^tg^Dek^−/−^ and Ron^tg^Dek^+/+^ females, which were continuously bred and monitored for tumor development. At necropsy, mammary tumors were excised and either fixed in 4% paraformaldehyde or dissociated to generate novel Ron^tg^Dek^−/−^ cell lines, which were also previously reported [51]. Usage and handling of mice was performed with the approval of the Cincinnati Children’s Institutional Animal Care and Use Committee under protocol 2017-0061. All mice were housed in specific pathogen free housing with ad libitum access to food and water.

### 4.2. Cell Culture

Cell lines RD147, RD238, and RD219 were generated by digesting tumor fragments in 2 mg/mL collagenase in DMEM:F12 media supplemented with 10% FBS, 1% penicillin-streptomycin, 1% Fungizone, 2 mM L-glutamine, 5 μg/mL gentamicin, 10 ng/mL EGF, 10 μg/mL human recombinant insulin, 5 μg/mL transferrin, and 50 μM sodium selenite then passaged at least 20 times, as previously described [51]. Cells were transduced with retroviral constructs, R780-Emtpy and R780-mDek, then sorted based on GFP expression. Cells were then adapted to grow in DMEM:F12 media supplemented with 10% FBS, 1% penicillin-streptomycin, 1% Fungizone, and 2 mM L-glutamine. To collect tumor conditioned media, transduced lines were seeded at 1000 cells/mL and kept in DMEM-F12 complete media for 48 h, then washed with 1× PBS and cultured for an additional 6 h with new DMEM-F12 complete media. After culturing, cancer cell conditioned media was transferred to a 50 mL conical tube, filtered through a 22 μM syringe filter, and added to media for bone marrow derived macrophages (BMDM) or collected for protein secretion analysis. After removing the tumor conditioned media (TCM), cultured tumors cells were collected for further analysis at the protein and RNA levels.

To supplement BMDM culture media with M-CSF, 5 × 10^5^ L929 cells were plated in T75 flasks incubated at 37 °C in 60 mL of DMEM:F12 media supplemented with 10% FBS, 1% penicillin-streptomycin, 1% Fungizone, and 2 mM L-glutamine (complete DMEM:F12-10). Conditioned media was collected from the L929 cells after 3 days in culture and filtered through a 22 μM filter bottle then frozen at −20 °C or used fresh in BMDM media.

Bone marrow-derived macrophages (BMDM) were generated from wild-type FVB/N mice as described previously [88]. FVB/N mice were chosen because this is the same genetic background as the murine breast cancer cells. For macrophages exposed to tumor conditioned media, macrophages were seeded at 10,000 cells/mL for 48 h. After 48 h, BMDM media was changed to 50% BMDM media and 50% filtered cancer cell conditioned media. Conditioned BMDM cells were collected after 24 h to explore RNA expression levels and after 1 h to explore protein levels.

### 4.3. RNA-Sequencing

Total RNA was isolated from RD147 cells transduced with R780-Empty retroviral vector or R780-mDek over-expressing vector using a Qiagen RNA extraction kit according to manufacturer’s instructions. 

Library Preparation and DNA Sequencing: 150 to 300 ng of total RNA determined by InvitrogenTM QubitTM high-sensitivity spectrofluorometric measurement was poly-A selected and reverse transcribed using Illumina’s TruSeq^®^ stranded mRNA library preparation kit. Each sample was fitted with one of 96 adapters containing a different 8 base molecular barcode for high level multiplexing. After 15 cycles of PCR amplification, completed libraries were sequenced on an Illumina HiSeq2500 in Rapid Mode, generating 20 million or more high quality 100 base long paired end reads per sample.

RNA-Seq Analysis: A quality control check on the fastq files was performed using FastQC. Upon passing basic quality metrics, the reads were trimmed to remove adapters and low-quality reads using default parameters in Trimmomatic1 [Version 0.33].

Alignment, Transcript Abundance and Differential Gene Expression Analysis: Quantification of mRNA expression levels was based on the TopHat/Cufflinks pipeline of the CCHMC DNA sequencing and Genotyping Core. Reads were aligned to the mouse mm10/GRCm38 reference genome using TopHat. The BAM files containing the aligned reads were used to quantify mRNA expression level using Cufflinks with the USCS known gene reference annotation. RNA expression values were normalized by the fragments per kilobase per megabase calculation (FPKM). Genes with an FPKM <0.4 were removed from the dataset to enrich for highly expressed genes and a list of differentially expressed genes was generated that were >1.5-fold up-regulated or <0.5-fold down-regulated. The subsequent list of all differentially expressed genes (both up- and down-regulated) were used to assess gene ontologies using the ToppFun program, which is part of the ToppGene Suite (https://toppgene.cchmc.org).

### 4.4. Quantitative RT-PCR

mRNA was extracted using the RNeasy Mini Kit (Qiagen) and 1 μg of RNA was reverse transcribed using the Quantitect Kit (Qiagen) to make cDNA. SYBR Green PCR master mix was used to amplify cDNA using an ABI-7500 quantitative PCR machine (Applied Biosystems, Foster City, CA, USA) and analyzed by the ΔΔCt method. Macrophages were collected 24 h after exposure to TCM. Primer sequences were used at a concentration of 0.4 ng/μL each and are listed in Table S2.

### 4.5. Western Blotting

Protein was extracted with NETN lysis buffer containing 200 mM Tris-HCl, 100 mM NaCl, 500 mM EDTA, 0.5% NP-40, 50 mM NaF, 0.2 mM Na_3_VO_4_ and 1× Protease Inhibitor Cocktail (Sigma-Aldrich, St. Louis, MO, USA). Fifty micrograms of protein was separated by SDS-PAGE gel and transferred to a PVDF membrane. Blots probed for phosphorylated proteins were blocked with 5% BSA in 1× TNET and all other probed proteins were blocked with 3% Milk and 5% BSA in 1× TNET. Blots were probed for the following antibodies: DEK (BD Bioscience, San Jose, CA, USA, #610948 or Proteintech/VWR, Rosemont, IL, USA, #10085-390), TSLP (eBioscience from Invitrogen, Waltham, MA, USA, #501122961), FPN1 (Novus Biologicals, Centennial, CO, USA, #NBP1-21502SS), Actin C4 (gift from James Lessard, Cincinnati Children’s Hospital Medical Center, available from Seven Hills Bioreagents, #LMAB-C4), phospho-ERK1/2, total ERK1/2 (Cell Signaling, Danvers, MA, USA, #9106 and #9102). Blots were imaged with ECL blotting reagent and the Bio-Rad ChemiDoc Imaging System, while densitometries were calculated via Bio-Rad Image Lab Software. For secreted proteins, 6.0 mL of conditioned media was collected from cancer cells and concentrated to 100 μL using Amicon Ultra-4 centrifugal filters with Ultracel-3 membrane (Millipore, Billerica, MA, USA) then 2 μL was diluted into 18 μL of serum free media prior to Western blotting.

### 4.6. Iron Assay

To analyze changes in iron retention, BMDMs were incubated in diluted tumor conditioned media (TCM) for 6 days and then treated with 10 µM ferric ammonium citrate (Sigma) overnight. Cells were lysed in the dish with EDTA-free NETN lysis buffer and 50 µL of lysate was used to determine intracellular iron content using the QuantiChrom Iron Assay Kit (BioAssay Systems, Hayward, CA, USA). Iron concentrations were normalized to the protein concentration of each sample.

### 4.7. Luminex Assay

1 × 10^5^ tumor cells were plated per well of a 24 well plate. Upon reaching confluency, complete media was replaced with serum free media. Six hours later, tumor-conditioned media was collected, centrifuged at 2000× *g* rpm for 10 min and supernatant was frozen at −80 °C. A multiplex Luminex assay (R&D Systems, Minneapolis, MN, USA) for mouse cytokines and chemokines was performed to test for the following cytokines were assayed: IL-6, MCP-1, GM-CSF, IL-4, IL-13, M-CSF, S100A8, and Lipocalin2. Manufacturer’s instructions were followed. Briefly, samples were mixed with 25 μL beads overnight. Plates were washed 2 times and then 25 μL detection antibody incubated for 1 h at room temperature. Then, 25 μL S-RPE was added directly to the detection antibody for 30 min. Plates were then washed again and then 150 μL sheath fluid added prior to analysis using a Luminex 200 dual-laser system. The assay was performed by the Cincinnati Children’s Hospital Medical Center Research Flow Cytometry Core.

### 4.8. Flow Cytometry

Flow cytometry was performed on a FACS Canto cytometer (BD Biosciences) and analyzed using Flo Jo software. After treatment with TCM, macrophages were removed from the plate with cell dissociation buffer then harvested in cold PBS, blocked with mouse CD16/CD32 Fc block (BD Biosciences) stained with the following fluorophore conjugated antibodies: F4/80-FITC (eBiosciences, San Diego, CA, USA), NOS2-PE-Cy7 (eBiosciences), ARG1-APC (R&D Systems). Samples were analyzed on a FACS Canto (BD Bioscences) and FlowJo software.

### 4.9. Immunohistochemistry and Immunofluorescence

Tissues were fixed in 4% paraformaldehyde and embedded in paraffin. Embedded tissues were cut into 5 μm sections. Slides were dewaxed and incubated in heated 10 mM sodium citrate prior to staining for antigen retrieval. Sections were manually stained for CD163 (Abcam, Cambridge, MA, USA, #ab182422, 1:50), TSLP (Clone: 28F12, eBioscience #501122961, 1:100), and F4/80 (eBioscience #311-4801, 1:50). Staining was completed with the M.O.M.^®^ (Mouse on Mouse) Elite^®^ Peroxidase Kit (#PK-2200) from Vector Laboratories and counterstained with Hematoxylin and Ammonia Hydroxide before preserving with Cytoseal (Thermofisher, Waltham, MA, USA) mounting media and coverslips. To quantify TSLP and CD163 positive staining, the ImageJ cell counter plugin was utilized to manually count each positive stained cell per field. A minimum of six fields were counted per tumor, and at least 3 tumors were analyzed per genotype.

For immunofluorescence, slides were stained for F4/80 (eBioscience #311-4801, 1:50) and FPN1 (Novus Biologicals, #NBP1-21502SS. 1:50) and counter-stained with Prolong Gold with DAPI (Invitrogen), then imaged on a Nikon C2 confocal microscope with Nikon Elements software.

### 4.10. Perl’s Prussian Blue Stain

Paraformaldehyde-fixed and paraffin-embedded tissues were cut into 5 μm sections, wax was removed by incubating a 60 °C oven for approximately 30 min, and slides were incubated 20 min in a working solution made of equal parts 5% potassium ferrocyanide and 5% hydrochloric acid. Tissues were counterstained 5 min in Nuclear Fast Red, then preserved with Cytoseal (Thermofisher) and coverslipped. Quantification of blue staining intensity was determined by separating the blue color channel using the ImageJ Colour Deconvolution plugin.

### 4.11. Human Breast Cancer Databases

SEMA: SEMA is a web-based program (https://sema.research.cchmc.org/) that creates graphical models using data from The Cancer Genome Atlas (TCGA). The user-defined model is then analyzed for fit to the data by Structural Equation Modeling (SEM) [89]. DEK mRNA levels were queried against the expression of target genes identified in the mouse model as well as “Tumor Features” from the PanCancer Atlas.

KM Plotter: The impact of combined DEK and CD163 mRNA expression for survival outcomes was analyzed using KM Plotter (https://kmplot.com/analysis/). The mRNA for breast cancer dataset was used, and the combination of DEK (Affy ID: 200934_at) and CD163 (Affy ID: 215049_x_at) were queried using the mean expression of each genes and the auto-select for best cut-off feature for their impact on categories of patient survival and intrinsic subtypes.

### 4.12. Statistics

An unpaired two-tailed Student’s *t*-test was used, unless otherwise noted. Error bars depict standard error of data collected from at least three experiments. Significance was set at *p* < 0.05. One asterisk (*) indicates *p* < 0.05, two asterisks (**) indicates *p* < 0.01, and three asterisks (***) indicates *p* < 0.001.

## 5. Conclusions

Several reports have focused on the intracellular oncogenic roles of DEK in solid tumor progression. Previous studies have shown that DEK acts as a transcription co-factor for NFkB, which would impact the expression of numerous cytokines and chemokines. We have demonstrated here that Dek-induced cytokine deregulation correlates with M2 TAM polarization. Dek also has also been shown to promote the production and secretion of Wnt ligands. Combined, this suggested that Dek expression could significantly impact the extracellular signaling molecules secreted by cancer cells, thus altering the tumor microenvironment. Here, for the first time, we utilize cell culture, mouse, and human dataset models to demonstrate that DEK expression in mammary tumors does, indeed, create a potentially pro-tumorigenic microenvironment through the M2 polarization of tumor associated macrophages. This is associated with worse overall survival outcomes, particularly in luminal subtypes of human breast cancer. Future work will focus on a more detailed view of the molecular mechanisms causing this microenvironment difference as well as the impact on response to therapies.

## Figures and Tables

**Table 1:** Selected up- and down-regulated genes in Dek expressing cells enriched for immunomodulatory genes and previously identified Dek target genes.

Gene	Fold Change	Gene	Fold Change
Dek	21.13	Hdac7	0.24
Cuedc2	12.95	Gas7	0.28
Rxrb	4.83	Cxcl2	0.31
Taz	3.24	Cxcl10	0.31
Hmga1	3.11	Setdb1	0.33
Tufm	2.91	Llgl1	0.36
Sphk2	2.67	Irf9	0.41
Rad54l/ATRX	2.29	Ccl17	0.41
Vegfa	2.11	Socs1	0.42
Tslp	1.84	Cxcl1	0.43
Tcf3	1.76	Ccl25	0.46
Cpeb2	1.59	Irf7	0.44
Cdc45	1.58	Cav2	0.46
Ercc1	1.57	Pias3	0.46
Gemin5	1.57	cdh1/E-cadherin	0.48
Ccl5	1.52	Rap1gap	0.48
Nfkb2	1.49	Casp8ap2	0.50

## Supplementary Materials

The following are available online at https://www.mdpi.com/2072-6694/12/7/1936/s1, Table S1: Differentially Expressed Genes, Table S2: Primer Sequences
