# Supplementary material for: DEK Expression in Breast Cancer Cells Leads to the Alternative Activation of Tumor Associated Macrophages
Source: Cancers (Basel). 2020 Jul 17;12(7):1936. doi: 10.3390/cancers12071936 (PMC7409092; doi:10.3390/cancers12071936)
Supplement: Supplementary file 1 [file cancers-12-01936-s001.zip › Supplementary Table S 2.docx]

Supplemental Materials: DEK Expression in Breast Cancer Cells Leads to the Alternative Activation of Tumor Associated Macrophages

Nicholas A. Pease, Miranda S. Shephard, Mathieu Sertorio, Susan E. Waltz and
Lisa M. Privette Vinnedge

**Table S2.** RT-PCR primer sequences.

| **Gene** | **Forward Primer (5’-3’)** | **Reverse Primer (5’-3’)** |
| --- | --- | --- |
| Actin | GATATCGCTGCGCTGGTCGTC | ACCATCACACCCTGGTGCCTAG |
| Arg1 | CTTCGGAACTCAACGGGAGG | TGTGATGCCCCAGATGGTTT |
| Ccl25 | AGTGTGTGGGAATCCAGAGG | TCCTCCAGCTGGTGCTTACT |
| Ccl5 | GTGCCCACGTCAAGGAGTAT | GCGGTTCCTTCGAGTGACAA |
| Cxcl1 | GCTGGGATTCACCTCAAGAA | AAGGGAGCTTCAGGGTCAAG |
| Dek | AACGTGGGTCAGTTCAGTGGC | TTCGCTGTTCACGCCTGACCT |
| Il-10 | AGAGGGTTCCCCTACTGTCA | TGGCCACAGTTTTCAGGGAT |
| Tnfa | ACCTGGCCTCTCTACCTTGT | CCCGTAGGGCGATTACAGTC |
| TSLP | GACAGCATGGTTCTTCTCAG | CTGGAGATTGACATGAAGG |
| Vegfa | TCTCCCAGATCGGTGACAGT | AAGGAATGTGTGGTGGGGAC |
| Ym1 | CTGGAATTGGTGCCCCTACAA | GGCATAGGGTACTTCCTGGG |

| 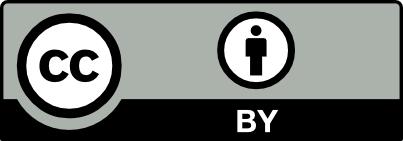 | © 2020 by the authors. Licensee MDPI, Basel, Switzerland. This article is an open access article distributed under the terms and conditions of the Creative Commons Attribution (CC BY) license (http://creativecommons.org/licenses/by/4.0/). |
| --- | --- |
